# Survival Outcomes in Patients with Squamous Cell Carcinoma of the Urinary Bladder: A Propensity Score-Matched Analysis

**DOI:** 10.3390/curroncol32070394

**Published:** 2025-07-10

**Authors:** Alper Coskun, Ahmet Bilgehan Sahin, Selva Kabul, Muhammed Abdurrahman Celik, Mursel Sali, Ender Eren Ozcelik, Adem Deligonul, Erdem Cubukcu, Meral Kurt, Gursel Savci, Turkkan Evrensel, Ismet Yavascaoğlu

**Affiliations:** 1 Department of Medical Oncology, Uludag University Faculty of Medicine, Bursa 16059, Turkey; absahin@uludag.edu.tr (A.B.S.); murselsali@uludag.edu.tr (M.S.); endereren@uludag.edu.tr (E.E.O.); ademd@uludag.edu.tr (A.D.); erdemcubukcu@uludag.edu.tr (E.C.); evrensel@uludag.edu.tr (T.E.); 2 Department of Pathology, Uludag University Faculty of Medicine, Bursa 16059, Turkey; selva@uludag.edu.tr; 3 Department of Internal Medicine, Uludag University Faculty of Medicine, Bursa 16059, Turkey; drmuhammedcellik@gmail.com; 4 Department of Radiation Oncology, Uludag University Faculty of Medicine, Bursa 16059, Turkey; mkurt@uludag.edu.tr; 5 Department of Radiology, Uludag University Faculty of Medicine, Bursa 16059, Turkey; gsavci@uludag.edu.tr; 6 Department of Urology, Uludag University Faculty of Medicine, Bursa 16059, Turkey; ismet@uludag.edu.tr

**Keywords:** bladder, cancer, propensity score matching, squamous cell carcinoma, survival

## Abstract

Background and Objective: Bladder cancer (BC) is the ninth most common malignancy worldwide. Squamous cell carcinoma (SqCC), a rare histological variant, accounts for approximately 2–5% of all BC cases. Compared to urothelial carcinoma, the predominant subtype, research on SqCC remains limited and shows inconsistent findings regarding prognosis. This study aimed to compare survival outcomes between patients with SqCC and those with pure urothelial carcinoma (PUC). Methods: This retrospective, observational study analyzed pathology reports from 2549 transurethral resections of bladder tumors and 632 cystectomies performed at our institution between 1 December 2010 and 31 December 2023. Following pathological re-evaluation, 33 patients with SqCC and 132 with PUC were identified. After 1:3 propensity score matching, 20 patients with SqCC and 58 with PUC were included in the final analysis. Demographic, clinicopathological features, and survival outcomes were compared between groups. Results: The median follow-up was 2.31 years (range: 0.17–13.50). No significant differences in baseline demographic or clinical characteristics were observed, except for the type of surgery. Kaplan–Meier analysis demonstrated no significant differences in disease-free survival (DFS; *p* = 0.961) or overall survival (OS; *p* = 0.847) between SqCC and PUC groups. Multivariate Cox regression analysis identified T stage, nodal involvement, and adjuvant chemotherapy (CT) as independent predictors of DFS, while sex and metastasis at diagnosis were significant predictors of OS. Conclusion: Survival outcomes (DFS and OS) did not significantly differ between patients with SqCC and patients with PUC. Prognosis was more closely associated with disease stage at diagnosis, sex, and adjuvant CT. Further large-scale studies are warranted.

## Simple Summary

Squamous cell carcinoma (SqCC) is a rare histological subtype of bladder cancer. Most studies in the literature focus on urothelial carcinoma, while studies on SqCC are fewer in number and generally based on small, retrospective cohorts. Unlike previous research, our study involved the pathological re-evaluation of all cases by a dedicated genitourinary pathologist, and patients with mixed or variant histologies were excluded. Additionally, we applied propensity score matching (PSM) to ensure comparable demographic and clinicopathological characteristics between the SqCC and pure urothelial carcinoma (PUC) groups. We believe that the inclusion of pathological re-evaluation and PSM adds value to our study. Our findings demonstrated that survival outcomes were similar between the SqCC and PUC groups. Furthermore, T stage, nodal involvement, and adjuvant chemotherapy were independent predictors of disease-free survival, while sex and metastasis at diagnosis were independent predictors of overall survival. Further large-scale studies are warranted in this area.

## 1. Introduction

Bladder cancer (BC) ranks as the ninth most common malignancy worldwide, according to 2022 data from the Global Cancer Observatory (GLOBOCAN) [1]. In that year, approximately 615,000 new cases were reported globally, with more than 220,000 associated deaths [2]. While various risk factors have been implicated in the development of BC, cigarette smoking remains the most significant risk factor in Western countries [3]. In contrast, in developing regions such as the Middle East and Africa, chronic infection with Schistosoma haematobium (schistosomiasis) is a major etiological factor [4].

The most common histopathological subtype of BC in developed nations is urothelial carcinoma (UC), accounting for over 90% of all cases [5]. Within the classification of BC, approximately 75% of patients present with pure urothelial carcinoma (PUC), whereas the remaining 25% exhibit variant histologies (VHs) [6]. These VHs include UC with divergent differentiation (e.g., squamous, neuroendocrine, mucinous, and nested) as well as non-urothelial carcinomas (NUCs) such as squamous cell carcinoma (SqCC), neuroendocrine carcinoma (NEC), and adenocarcinoma. A large-scale retrospective analysis comparing UC and NUC cases reported that non-squamous histologies occurred more frequently in males, and NUCs were more often diagnosed at advanced stages. In contrast, UC was typically diagnosed at earlier stages. Furthermore, among the non-urothelial subtypes, SqCC and NEC were associated with significantly worse prognoses compared to UC [7].

SqCC is recognized as the second most common histological subtype of BC after UC [8]. SqCC develops through a process of squamous metaplasia, in which the transitional epithelium of the bladder undergoes chronic irritation, leading to the replacement of urothelial cells with squamous epithelium [9]. While SqCC accounts for approximately 2–5% of BC cases in Western countries, its incidence is significantly higher in developing regions such as the Middle East and Africa. This increased prevalence is strongly associated with Schistosoma haematobium infection, which is considered the leading risk factor for SqCC and accounts for up to 80% of BC cases in Egypt [4,10]. In contrast, in developed countries, the primary risk factors for SqCC include recurrent urinary tract infections and long-term catheter use [9]. Histologically, SqCC is characterized by full squamous differentiation, including intercellular bridges and keratinization [11]. Unlike squamous differentiation in urothelial carcinoma (SD), which contains partial or focal areas of squamous features, SqCC exhibits 100% squamous differentiation with more pronounced histological characteristics [12]. Clinically, SqCC is associated with a higher rate of locoregional recurrence compared to UC, and this is considered the primary cause of mortality in patients with SqCC [13,14].

The aim of this study was to evaluate the clinical characteristics and survival outcomes of patients diagnosed with SqCC in comparison to those with PUC. Given the rarity of SqCC—comprising less than 5% of BC cases—and the limited number of studies available, this study seeks to provide valuable insights into its clinical behavior and prognosis, thereby contributing to the existing literature.

## 2. Materials and Methods

### 2.1. Study Design

This study is a clinical, retrospective, observational analysis conducted at Bursa Uludag University Faculty of Medicine Hospital. The pathology reports of all transurethral resections of bladder tumors (TURBT; *n* = 2549) and cystectomies (*n* = 632) performed between 1 December 2010 and 31 December 2023 were reviewed.

### 2.2. Study Population

Demographic and clinical data were obtained for patients aged 18 years and older (*n* = 33) who were diagnosed with SqCC in either TURBT or cystectomy pathology reports or who had SqCC among the possible differential diagnoses.

After histopathological re-evaluation (described below), 22 patients were confirmed to have SqCC and were included in the SqCC group. Additionally, pathology records of 132 patients previously diagnosed with PUC were re-evaluated. Upon re-evaluation, two cases were re-classified as SqCC, five as SD, one as neuroendocrine differentiation, and one as mucinous differentiation. Patients with VHs (*n* = 7) were excluded, while the two newly identified SqCC cases were added to the SqCC group, bringing the total to 24 SqCC patients.

**Figure 1:**
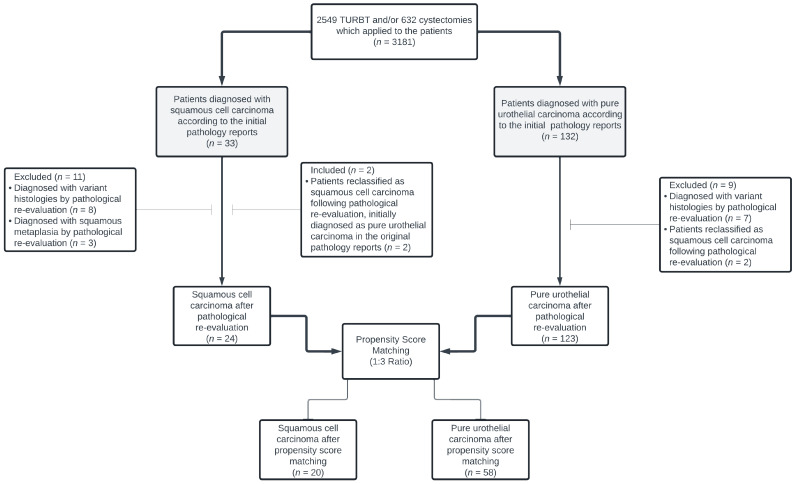
The study flow diagram.

In total, 147 patients (24 with SqCC and 123 with PUC) were evaluated for propensity score matching (PSM). PSM was conducted at a 1:3 ratio based on age, sex, tumor-node-metastasis (TNM) stage, neoadjuvant and adjuvant chemotherapy (CT), and follow-up duration. This process resulted in 20 SqCC patients matched with 58 PUC patients, yielding a final study population of 78 patients. A flowchart illustrating the patient selection process is shown in Figure 1. Standardized mean differences (SMDs) for the six covariates evaluated for matching were calculated for both the SqCC and PUC groups, before and after PSM. Detailed SMD results are presented in Supplementary Table S1.

### 2.3. Histopathological Evaluation

All available pathology slides were re-evaluated by a dedicated genitourinary pathologist to ensure diagnostic accuracy and to exclude VHs. This reassessment played a critical role in refining both the SqCC and PUC cohorts.

### 2.4. Data Collection

Data were collected via the hospital automation system and retrospective chart review. Variables included general demographic characteristics, pathological diagnoses, comorbid conditions, smoking history, TNM stage at diagnosis, presence and location of metastasis, details of neoadjuvant and adjuvant treatment (including CT protocols and cisplatin use), treatment response, disease progression, recurrence patterns, and survival outcomes.

### 2.5. Survival Definitons

In our study, disease-free survival (DFS) was defined as the time from curative surgery to either tumor recurrence, death, or last follow-up. Progression-free survival (PFS) was defined as the time from initiation of systemic treatment for metastatic disease to disease progression, death, or last follow-up. Overall survival (OS) was defined as the time from diagnosis to death or last follow-up.

### 2.6. Statistical Analysis

All statistical analyses were performed using IBM SPSS Statistics version 25.0. PSM was conducted using NCSS 2025 software. Categorical variables are presented as frequencies and percentages, while continuous variables are reported as means, standard deviations, medians, and minimum–maximum values. Normality of distribution was assessed using the Kolmogorov–Smirnov and Shapiro–Wilk tests. For comparisons between two groups, the independent samples *t*-test was used for normally distributed variables, while the Mann–Whitney U test was used for non-normally distributed variables. Survival analyses were performed using the Kaplan–Meier method, and survival curves were generated accordingly. Univariate and multivariate Cox proportional hazards regression models were used to identify factors independently associated with survival outcomes. Prior to PSM, binary logistic regression analysis was conducted using SPSS to calculate predictive values, and matching was subsequently performed with NCSS 2025. A *p*-value of <0.05 was considered statistically significant throughout the analysis.

## 3. Results

A total of 78 patients were included in the study following 1:3 PSM. Of these, 20 patients (25.6%) were diagnosed with SqCC and 58 patients (74.4%) with PUC. The median age of the cohort was 66.0 years (range: 48–90), and seven patients (9%) were female. The median follow-up period was 2.31 years (range: 0.17–13.50). Hypertension was the most common comorbidity, observed in 42 patients (53.8%). There were no statistically significant differences between the SqCC and PUC groups in terms of demographic or clinical characteristics, except for the type of surgery performed (radical vs. partial). Detailed patient characteristics are presented in Table 1.

Among patients who were non-metastatic at diagnosis (17 SqCC and 48 PUC), recurrence occurred in 10 (58.8%) of the SqCC group and 29 (60.4%) of the PUC group. Except for one PUC patient who developed a locoregional recurrence, all recurrences were metastatic. Lung metastasis was most frequently observed in the SqCC group (four patients, 23.5%), whereas bone metastasis was the most common in the PUC group (six patients, 12.5%), followed by lung metastasis in an additional six patients (12.5%). Cerebral metastasis was not observed in either group. Liver metastasis was reported in one patient from each group (5.9% in SqCC; 2.1% in PUC).

**Figure 2:**
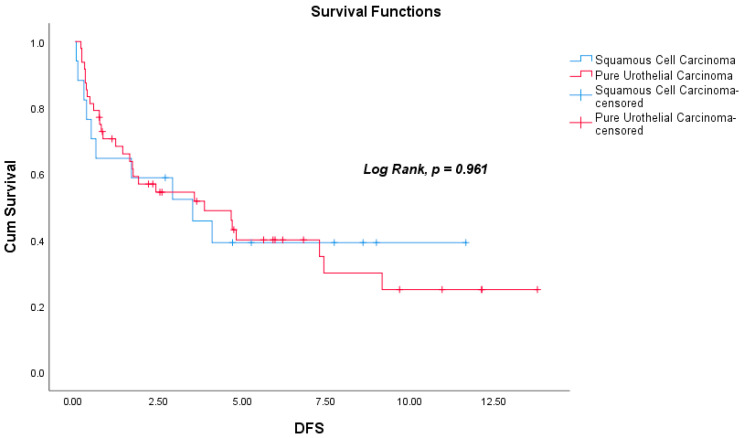
Kaplan–Meier survival plot for disease-free survival (DFS, Log-Rank, *p* = 0.961).

**Figure 3:**
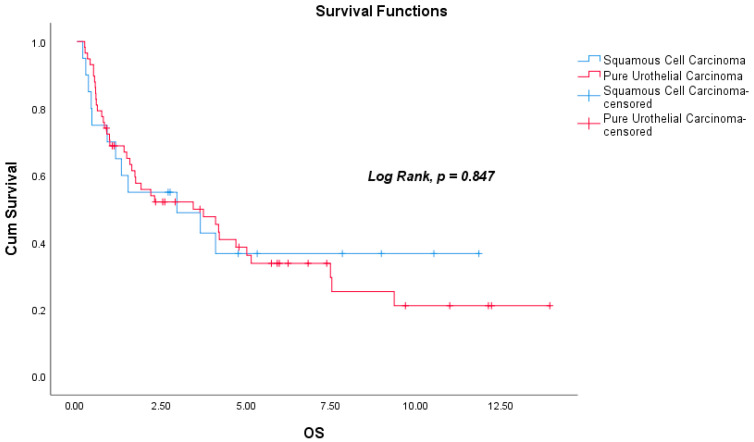
Kaplan–Meier survival plot for overall survival (OS, Log-Rank, *p* = 0.847).

**Figure 4:**
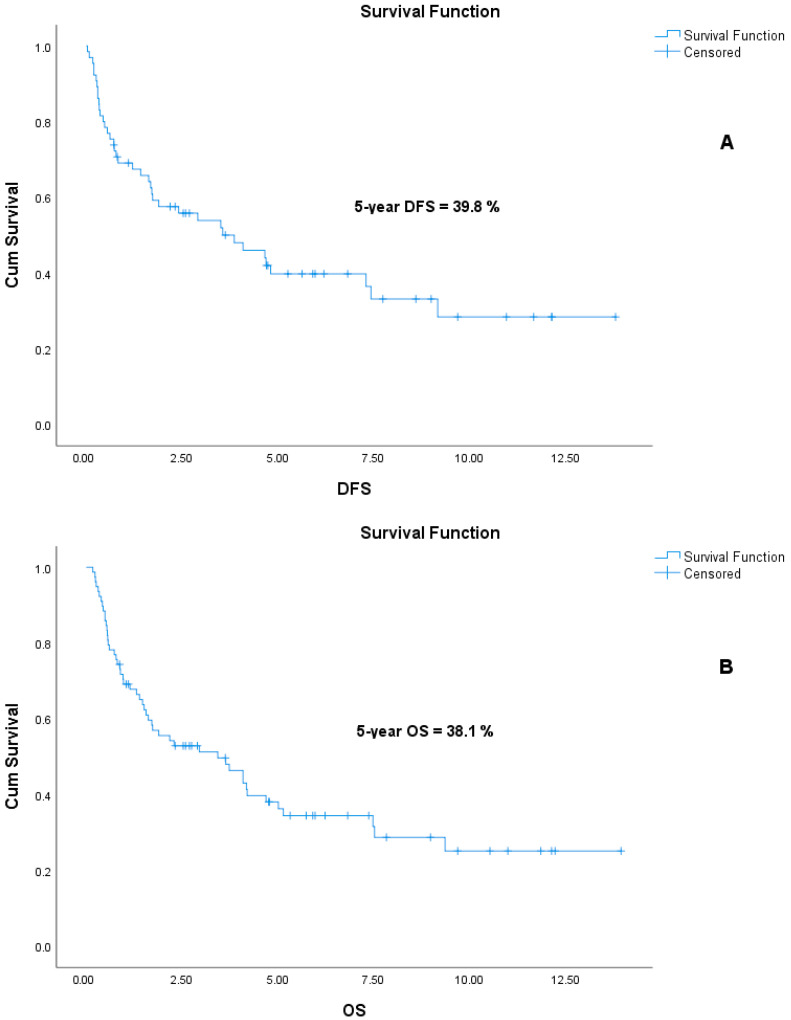
Kaplan–Meier survival curves illustrating actuarial five-year disease-free survival (DFS) (**A**) and overall survival (OS) (**B**) rates.

During the follow-up period, death occurred in 12 patients (60%) in the SqCC group and in 38 patients (65.5%) in the PUC group. Among the 65 patients who were non-metastatic at diagnosis, the median DFS was 2.41 years (range: 0.03–13.81), and the median OS was 2.90 years (range: 0.17–13.95). The median DFS was 2.91 years (range: 0.03–11.67) in the SqCC group and 2.26 years (range: 0.17–13.81) in the PUC group (Log-Rank, *p* = 0.961). Median OS for the entire cohort was 2.31 years (range: 0.17–13.95); subgroup analysis revealed a median OS of 2.72 years (range: 0.17–11.86) for SqCC patients and 2.24 years (range: 0.23–13.95) for PUC patients (Log-Rank, *p* = 0.847). There were no significant differences in DFS or OS between the two histological groups. Kaplan–Meier survival curves for DFS and OS are shown in Figure 2 and Figure 3, respectively. In addition, the actuarial five-year DFS rate was 39.8%, and the actuarial five-year OS rate was 38.1%. Kaplan–Meier survival curves for five-year DFS and OS are presented in Figure 4.

Univariate and multivariate Cox regression analyses were performed to identify factors influencing DFS and OS. Diagnostic T stage, nodal involvement at diagnosis, and receipt of adjuvant CT were found to be independent predictors of DFS. Sex and metastasis at diagnosis were identified as independent predictors of OS. Detailed results of the regression analyses are provided in Table 2 and Table 3.

At the time of diagnosis, 13 patients (16.7%) were found to have metastatic disease—three in the SqCC group (15%) and 10 in the PUC group (17.2%). Lung metastasis was the most common site in metastatic patients, present in six individuals (one SqCC, five PUC). Eleven of the thirteen metastatic patients (84.6%) received first-line systemic CT (two SqCC, nine PUC). CT regimens included cisplatin + gemcitabine (seven patients), carboplatin + gemcitabine (three patients), and carboplatin + paclitaxel (one patient). The median duration of first-line CT in the metastatic setting was 3.53 months (range: 0.93–34.83).

During follow-up, 17 patients who were initially non-metastatic developed metastatic disease. Of these, 11 patients (64.7%) received first-line systemic CT. The administered regimens included carboplatin + paclitaxel (six patients), cisplatin + gemcitabine (three patients), carboplatin + gemcitabine (one patient), and gemcitabine monotherapy (one patient). Among these patients, the median PFS following first-line CT was 4.8 months (range: 0.66–18.26). When all patients who received metastatic first-line treatment were considered, the median PFS was 3.65 months (range: 0.66–34.83). The median number of treatment cycles was three (range: 1–10). Disease progression occurred in all patients, and no treatment-limiting toxicities were reported. None of the patients received immune checkpoint inhibitors (ICIs) or antibody-drug conjugates (ADCs) as part of first-line or maintenance therapy.

Second-line metastatic treatment was administered to 12 patients (three SqCC, nine PUC). Treatment regimens included carboplatin + paclitaxel (four patients), docetaxel monotherapy (three patients), paclitaxel monotherapy (two patients), cisplatin + gemcitabine (one patient), vinflunine (one patient), and atezolizumab (one patient). The median number of second-line treatment cycles was four (range: 1–12). At the time of analysis, two patients receiving docetaxel monotherapy were still under treatment without evidence of disease progression. The remaining 10 patients experienced disease progression. No patients received third-line systemic treatment.

## 4. Discussion

In our study, no statistically significant difference was observed in survival outcomes when patients diagnosed with SqCC were compared to those diagnosed with PUC, matched in a 1:3 ratio using PSM. Multivariate Cox regression analysis demonstrated that diagnostic T stage, nodal involvement at diagnosis, and receipt of adjuvant CT were independent risk factors affecting DFS, while sex and metastasis at diagnosis were independent risk factors influencing OS.

Numerous clinical studies have compared SqCC and UC in the literature. However, their survival outcomes remain inconsistent. While some studies report a poorer prognosis for SqCC compared to UC [8,15,16], others show similar survival outcomes between the two subtypes [12,17,18]. One study even suggests comparable early-stage survival, with poorer outcomes for SqCC at advanced stages [19].

In our study, patients with SqCC and PUC were comparatively evaluated following pathological re-evaluation, excluding patients with VHs. This re-evaluation was conducted by reviewing both initial pathology reports and reassessment by expert pathologists. Notably, in a study involving 589 TURBT specimens reviewed by genitourinary pathologists, the presence of VHs had been missed in 44% of cases by the initial pathologists [20]. These findings emphasize the importance of pathology review by experienced genitourinary specialists in ensuring accurate diagnosis and guiding appropriate management. It is noteworthy that studies incorporating systematic pathological re-evaluation in BC remain scarce [20,21,22,23,24], and, to our knowledge, no other study has applied this approach specifically to SqCC patients.

In a Surveillance, Epidemiology, and End Results (SEER)-based study analyzing data from 2004 to 2016, SqCC was identified as the histological subtype with the poorest prognosis across all stages. Other VHs, including NEC and adenocarcinoma, also had worse outcomes than UC in non-metastatic cohorts [8]. Another large SEER-based study evaluating 394,979 BC cases (4783 with SqCC) with clinical staging T2–T4N0M0 demonstrated that SqCC was associated with significantly poorer survival across stages [15]. Similarly, in a United States (U.S.) national cancer database study using 1:1 PSM to compare SqCC and UC patients treated with chemoradiotherapy, SqCC was linked to worse OS (median OS: 15.1 vs. 30.4 months, *p* = 0.013). Cox regression analysis in that study identified pathological subtype, age > 75, Charlson–Deyo comorbidity index, and T stage as independent risk factors for OS [16]. A retrospective study by Agrawal et al. comparing 37 SqCC, 89 SD, and 908 PUC patients who underwent radical cystectomy reported inferior survival in SD patients, with no significant difference between SqCC and PUC in terms of relapse-free survival (RFS), cancer-specific survival (CSS), and OS, consistent with our findings [12]. Another study assessing 60 SqCC and 364 UC patients found no survival difference between the subtypes when adjusted for stage. The study emphasized the prognostic significance of nodal involvement, which is aligned with our finding that nodal involvement independently influenced DFS [17]. Similarly, another study reported no significant differences in treatment response (27% in SqCC vs. 44% in PUC) or OS (13.6 vs. 11.3 months) [18]. Conversely, a different study reported that SqCC was an independent adverse prognostic factor in stage I–II and stage III–IV BC patients who did not undergo cystectomy; however, no survival difference was observed in early-stage and surgically treated cases [19].

Adjuvant cisplatin-based CT has been shown to improve survival outcomes (RFS, metastasis-free survival [MFS], and OS) in muscle-invasive bladder cancer (MIBC) patients in a systematic review of 10 randomized controlled trials (RCTs) [25]. Another study demonstrated superior OS in patients receiving adjuvant CT—especially among SqCC, sarcomatoid, and micropapillary subtypes—compared to those who did not receive it [26]. A meta-analysis in node-positive or T3–T4 BC patients undergoing radical cystectomy revealed better RFS, CSS, and OS with adjuvant cisplatin-based CT compared to non-cisplatin-based regimens [27]. Similarly, another meta-analysis of four RCTs indicated improved PFS and OS with adjuvant CT in MIBC patients [28].

Sex-related disparities in survival have also been reported. A study by Zhao et al. found worse OS in female patients with BC compared to males, with poorer DFS in women with body mass index (BMI) ≤ 24 but no difference in those with BMI > 24 [29]. A meta-analysis further revealed inferior CSS and OS in female BC patients, especially in muscle-invasive disease, with similar outcomes in non-muscle-invasive cases [30]. Another study suggested that women had a worse response to intravesical Bacillus Calmette–Guérin (BCG) therapy in non-invasive BC [31]. In our study, female sex was identified as an independent adverse prognostic factor for OS, consistent with these findings [29,30,31].

Evidence suggests that SqCC responds poorly to neoadjuvant cisplatin-based CT compared to UC. However, therapies targeting epidermal growth factor receptor (EGFR) may enhance neoadjuvant CT efficacy in SqCC [15,32,33,34,35]. Due to the limited number of patients receiving neoadjuvant CT in our study (*n* = 8, with only two in the SqCC group), we were unable to conduct meaningful comparative analysis. Moreover, four of the eight patients received carboplatin-based regimens instead of the standard cisplatin, further limiting interpretation. Accordingly, neoadjuvant CT did not show a survival benefit in our cohort.

In the treatment of BC, the use and importance of modern systemic therapies, including ICIs and ADCs, have increased significantly in recent years. These agents are particularly preferred in patients with PUC, and several retrospective clinical studies in the literature have compared their efficacy between VHs and PUC [36,37,38,39]. In a study evaluating BC patients treated with pembrolizumab (an anti–PD-1 ICI), response rates were similar between patients with SD and those with PUC; however, patients with sarcomatoid differentiation demonstrated better responses compared to the PUC group [36]. Another study involving patients with advanced BC and upper-tract urothelial carcinoma treated with pembrolizumab reported comparable treatment responses between VHs and PUC [37]. In a cohort of metastatic SD and PUC patients treated with enfortumab vedotin (an anti–nectin-4 ADC) or ICIs, survival outcomes were found to be worse in SD patients compared to those with PUC [38]. Similarly, in another study comparing patients with VHs and PUC treated with enfortumab vedotin, PFS was worse in the VHs group, whereas OS was similar between the two groups [39]. To date, there are no prospective or retrospective clinical studies specifically evaluating the use of these agents in patients with SqCC. In our study, only one patient in the metastatic setting received ICI therapy, and no patient received ADC therapy; therefore, we were unable to assess the impact of these treatments on survival outcomes. Given the growing role of ICIs and ADCs in oncology practice, there is a clear need for larger studies to further investigate their efficacy in patients with VHs, including SqCC.

This study has several limitations. Primarily, it is a retrospective, single-center study with a relatively small sample size. We would like to emphasize that the statistical power of our study is limited due to the small sample size. Additionally, the limited number of patients receiving neoadjuvant CT (*n* = 8) and adjuvant radiotherapy (*n* = 3) precluded a robust assessment of their survival effects. A further limitation relates to the unavailability of modern systemic therapies—such as ICIs, ADCs, and erdafitinib—as first-line, adjuvant, or subsequent-line therapies due to reimbursement restrictions in our country. As a result, most patients did not receive these treatments.

## 5. Conclusions

In conclusion, our study found no significant differences in DFS or OS between patients with SqCC and those with PUC. While survival outcomes in the literature remain variable, our findings align with several previous studies. Regardless of pathological subtype, factors such as sex, T stage, nodal involvement, metastasis at diagnosis, and receipt of adjuvant CT were identified as significant predictors of survival (DFS and OS), consistent with existing evidence. These results highlight the prognostic importance of disease burden and treatment status over histological subtype. There remains a need for multicenter, large-scale studies to further characterize the clinicopathological and molecular features of SqCC and to guide optimal management strategies.

## Figures and Tables

**Table 1:** The general characteristics of the patients.

Characteristics (*N* = 78)	SqCC (*n* = 20)	PUC (*n* = 58)	*p*
Age, Median (Min–Max)	65.0 (53.0–84.0)	66.5 (48.0–90.0)	0.948 *
Gender			0.853 **
Male	18 (90%)	53 (91.4%)	
Female	2 (10%)	5 (8.6%)	
Smoking Status			0.846 **
Yes	6 (30%)	21 (36.2%)	
No	1 (5%)	5 (8.6%)	
Smoking (pack/year) (mean ± SD)	42.6 ± 16.6	37.0 ± 22.4	0.613 *
Diagnosis T Stage			0.080 **
T1-T2 ^a^	9 (45%)	14 (24.1%)	
T3-T4 ^b^	11 (55%)	44 (75.9%)	
Diagnosis N Stage			0.954 **
Node negative	14 (70%)	41 (70.7%)	
Node positive	6 (30%)	17 (29.3%)	
Diagnosis M Stage			0.818 **
M0	17 (85%)	48 (82.8%)	
M1	3 (15%)	10 (17.2%)	
Diagnosis TNM Stage			0.856 **
Stage II	5 (25%)	14 (24.1%)	
Stage III	12 (60%)	34 (58.6%)	
Stage IV	3 (15%)	10 (17.2%)	
Neoadjuvant CT	2 (10%)	6 (10.3%)	0.937 **
Primary Surgery Status			0.976 **
Yes	19 (95%)	55 (94.8%)	
No	1 (5%)	3 (5.2%)	
Type of Surgery			0.021 **
RC	16 (80%)	54 (93.1%)	
PC	3 (15%)	1 (1.7%)	
Adjuvant CT	5 (25%)	14 (24.1%)	0.985 **
Follow-up (year) (mean ± SD)	3.59 ± 3.60	3.51 ± 3.46	0.877 **

* Independent samples *t*-test. ** Mann–Whitney U test. ^a^ SqCC, T1 (*n* = 2), T2 (*n* = 7); PUC, T2 (*n* = 14). ^b^ SqCC, T3 (*n* = 9), T4 (*n* = 2); PUC, T3 (*n* = 29), T4 (*n* = 15). CT, chemotherapy; PC, partial cystectomy; PUC, pure urothelial carcinoma; RC, radical cystectomy; SqCC, squamous cell carcinoma; TNM, tumor, node, metastasis.

**Table 2:** Univariate and multivariate Cox regression analyses for DFS.

Factor	DFS							
	Univariate	%95 CI		*p*	Multivariate	%95 CI		*p*
	HR	Lower	Upper		HR	Lower	Upper	
Age ^a^	1.292	0.641	2.607	0.474				
Gender ^b^	0.227	0.084	0.614	0.003	0.663	0.231	1.902	0.445 *
Smoking status ^c^	0.785	0.219	2.820	0.711				
Smoking (pack/year)	1.006	0.975	1.038	0.713				
Pathological subtype ^d^	1.018	0.495	2.093	0.961				
Diagnosis T stage ^e^	2.059	0.975	4.351	0.058	4.026	1.719	9.428	0.001 *
Diagnosis nodal status ^f^	2.039	1.028	4.046	0.042	3.128	1.462	6.691	0.003 *
Diagnosis TNM stage ^g^								
Stage III	2.325	1.023	5.281	0.044				
Neoadjuvant CT ^h^	2.423	1.063	5.524	0.035				
Neoadjuvant CT Cisplatin status ^i^	0.712	0.156	3.241	0.660				
Neoadjuvant CT Number of cycles	1.521	0.535	4.320	0.431				
Surgery type ^j^	3.805	1.314	11.021	0.014				
Adjuvant CT ^k^	0.249	0.105	0.590	0.002	0.120	0.045	0.317	0.000 *
Adjuvant CT cisplatin status ^l^	0.379	0.089	1.606	0.188				
Adjuvant CT number of cycles	1.022	0.590	1.771	0.938				

Reference groups: ^a^ Younger than 65 years old; ^b^ female sex; ^c^ no smoking; ^d^ squamous cell carcinoma; ^e^ T1-T2 stage at diagnosis; ^f^ node negative at diagnosis; ^g^ stage II at diagnosis; ^h^ no neoadjuvant CT; ^i^ no cisplatin in neoadjuvant CT; ^j^ RC; ^k^ no adjuvant chemotherapy; ^l^ no cisplatin in adjuvant CT. CT, chemotherapy; DFS, disease-free survival; RC, radical cystectomy; TNM, tumor, node, metastasis. * Enter method.

**Table 3:** Univariate and multivariate Cox regression analyses for OS.

Factor	OS							
	Univariate	%95 CI		*p*	Multivariate	%95 CI		*p*
	HR	Lower	Upper		HR	Lower	Upper	
Age ^a^	1.268	0.699	2.302	0.434				
Gender ^b^	0.283	0.116	0.692	0.006	0.389	0.154	0.986	0.047 *
Smoking status ^c^	0.853	0.246	2.958	0.802				
Smoking (pack/year)	0.992	0.968	1.016	0.509				
Pathological subtype ^d^	1.066	0.557	2.042	0.847				
Diagnosis T stage ^e^	2.162	1.078	4.333	0.030	1.574	0.761	3.256	0.221 *
Diagnosis nodal status ^f^	2.533	1.425	4.504	0.002	1.652	0.870	3.137	0.125 *
Diagnosis metastasis status ^g^	4.797	2.415	9.527	0.000	3.533	1.699	7.344	0.001 *
Diagnosis TNM stage ^h^				0.000				
Stage III	2.109	0.925	4.811	0.076				
Stage IV	8.466	3.227	22.214	0.000				

Reference groups: ^a^ Younger than 65 years old; ^b^ female sex; ^c^ no smoking; ^d^ squamous cell carcinoma; ^e^ T1-T2 stage at diagnosis; ^f^ node negative at diagnosis; ^g^ no metastasis at diagnosis; ^h^ stage II at diagnosis. OS, overall survival; TNM, tumor, node, metastasis. * Enter method.

## Data Availability

Data will be available from the corresponding author upon reasonable request.

## Supplementary Materials

The following supporting information can be downloaded at: https://www.mdpi.com/article/10.3390/curroncol32070394/s1, Supplementary Table S1: Standardized mean differences (SMDs) for all covariates before and after matching.
