# Supplementary material for: Survival Outcomes in Patients with Squamous Cell Carcinoma of the Urinary Bladder: A Propensity Score-Matched Analysis
Source: Curr Oncol. 2025 Jul 10;32(7):394. doi: 10.3390/curroncol32070394 (PMC12293893; doi:10.3390/curroncol32070394)
Supplement: Supplementary file 1 [file curroncol-32-00394-s001.zip › curroncol-3742594-supplementary.pdf]

Supplementary Table S1. Standardized mean differences (SMDs) for all covariates before and after matching.

Covariates	SMD (before PSM)	SMD (after PSM)
**Age**	0.08	0.02
**Sex**	0.91	0.16
**Diagnosis TNM Stage**		
**Stage I**	0.48	-
**Stage II**	0.09	0.04
**Stage III**	0.03	0.02
**Stage IV**	0.17	0.14
**Neoadjuvant CT**	0.35	0.03
**Adjuvant CT**	0.56	0.04
**Follow-up**	0.09	0.02

CT, chemotherapy; PSM, propensity score matching; TNM, tumour, node, metastasis
